# Family-based case-control study of exposure to household humidifier disinfectants and risk of idiopathic interstitial pneumonia

**DOI:** 10.1371/journal.pone.0221322

**Published:** 2019-09-05

**Authors:** Dirga Kumar Lamichhane, Jong-Han Leem, Sang-Min Lee, Hyeon-Jong Yang, Jaiyong Kim, Jong-Hyun Lee, Jung Keun Ko, Hwan Cheol Kim, Dong-Uk Park, Hae-Kwan Cheong

**Affiliations:** 1 Department of Social and Preventive Medicine, College of Medicine, Inha University, Incheon, Korea; 2 Department of Occupational and Environmental Medicine, College of Medicine, Inha University, Incheon, Korea; 3 Division of Pulmonary and Critical Care Medicine, Department of Internal Medicine, Seoul National University, College of Medicine, Seoul, Korea; 4 Department of Pediatrics, Soonchunhyang University Hospital, Soonchunhyang University, College of Medicine, Seoul, Korea; 5 Health and Society Institute, Hanyang University, College of Medicine, Seoul, Korea; 6 Environmental Health R & C, Bucheon, Korea; 7 Department of Environmental Health, Korea National Open University, Seoul, Korea; 8 Department of Social Medicine, Sungkyunkwan University School of Medicine, Suwon, Korea; Universite de Bretagne Occidentale, FRANCE

## Abstract

**Background:**

In Korea, several household humidifier disinfectants (HDs) were clinically confirmed to cause HD-associated lung injury (HDLI). Polyhexamethylene guanidine (PHMG) phosphate is the main ingredient of the HDs found to be associated with lung disease. However, the association of HDs with other interstitial lung disease including idiopathic interstitial pneumonia (IIP) is not clear. We examined the relationship between HD exposure and IIP in a family-based study.

**Methods:**

This case-control study included 244 IIP cases and 244 family controls who lived with the IIP patients. The IIP cases were divided into two groups, HDLI and other IIP, and were matched to family controls based on age and gender. Information on exposure to HDs was obtained from a structured questionnaire and field investigations. Conditional logistic regression was used to estimate odds ratio (ORs) and their corresponding 95% confidence interval (CI), investigating the association of HD-related exposure characteristics with IIP risk.

**Results:**

The risks of IIP increased two-fold or more in the highest compared with the lowest quartile of several HD use characteristics, including average total use hours per day, cumulative sleep hours, use of HD during sleep, and cumulative exposure level. In analyses separated by HDLI and other IIP, the risks of HDLI were associated with airborne HD concentrations (adjusted OR = 3.01, 95% CI = 1.34–6.76; Q_4_ versus Q_1_) and cumulative exposure level (adjusted OR = 3.57, 95% CI = 1.59–8.01; Q_4_ versus Q_1_), but this relationship was not significant in the patients with other IIP. In comparison between HDLI and other IIP, the odds ratios of average total use hours, cumulative use hours, and cumulative sleeps hours was higher for other IIP.

**Conclusion:**

The use of household HDs is associated not only with HDLI but also with other IIP.

## Introduction

Several types of humidifier disinfectants (HDs) have been available in South Korea since 1994 for the prevention of microbial contamination in humidifiers. However, the Korean Centers for Disease Control and Prevention (KCDC) has declared HDs as a cause of lung injury and the use of all HDs has been banned since 2011 [1]. Several studies have demonstrated that HDs were associated lung injury, including interstitial pneumonitis and widespread lung fibrosis [2–6]. Recent studies suggested that the risks of development of HD-associated lung injury (HDLI) were linked to the specific disinfectant exposure-related characteristics in a dose-dependent manner [7–9].

In South Korea, HDLI Investigation and Decision Committee (HLIIDC) officially investigated the registered patients with lung injuries through the various phases of investigation and determined whether the injuries were clinically associated with HD use [8]. According to the defined criteria [10], the victims were identified as a lung injury caused by HD and were classified as HDLI and other idiopathic interstitial pneumonia (IIP). Among the total of 530 registered victims in the first and second rounds of the HLIIDC, approximately 47% from the first investigation and 29% from the second investigation were confirmed as HDLI and the rest were classified as other IIP [10]. The government provides compensation for those patients whose conditions were confirmed to have been associated with the use of HDs. The HDLI patients included victims from all age groups, ranging from fetuses, children, pregnant women, and elderly patients aged >80 years [9]. A nationwide study among general population revealed that approximately 30% of Korean children were exposed to HD, and most of them (approx. 58%) used HD for less than three months [11]. Likewise, a study among a general population of adults in Gyeonggi province (Korea) reported that 37.2% of the 94 subjects used a humidifier, and the rate of HD usage was 18.1% [12]. Another study reported that 45.5% of pregnant women used a humidifier during the winter season [13].

Commonly used HDs, in particular, polyhexamethylene guanidine phosphate (PHMG) and oligo (2-(2-ethoxy) ethoxyethyl guanidinium (PGH), have been associated with HDLI [2–4, 14]. Airborne HD concentration, number of HD brands, duration of HD use, and cumulative HD levels have also been reported to be risk factors for the development of HDLI [7–9]. So far three case-control studies have investigated the dose-response relationship between HD exposure-related risk factors and HDLI [7, 9, 15]. However, these studies did not examine the relationship of the risk factors for other interstitial lung diseases. We thus conducted this case-control study to examine the relationship between HD exposure-related characteristics and risk of IIP, a general category that includes many different lung conditions including HDLI.

## Materials and methods

### Study design and subjects

We used data from the Korea Centers for Disease Control and Prevention (KCDC). The KCDC officially collected information on individuals with lung disease who presumed that their disease was related to humidifier disinfectant use. As previously reported [7], the HLIIDC has officially initiated the investigation of damage survey of the HDs in July 2013. This investigation committee consisting of pediatric pulmonologists, adult pulmonologists, radiologists, and pathologists evaluated the degree of damage based environmental exposure, histopathology, radiology, and clinical tests. All registered subjects were clinically examined by the committee to diagnose and confirm IIP including HDLI. A combination of clinical manifestations, natural disease course, and radiological and pathological findings was used to confirm the diagnosis [3, 16]. For this study, IIP was defined as those with Korean Classification of Disease 7^th^ revision (KCD-7) code of K-J84.0 (alveolar and parietoalveolar conditions), K-J84.10 (lymphoid interstitial pneumonia), K-J84.18 (other interstitial pulmonary disease with fibrosis), K-J84.8 (other specified interstitial pulmonary diseases) and K-J84.9 (interstitial pulmonary disease, unspecified). Korea has developed the KCD-7 code based on International Classification of Disease 10^th^ revision (ICD-10) code [17]. In this study, the IIP patients were divided into two groups: clinically confirmed HDLI patients and patients with other IIP.

**Fig 1:**
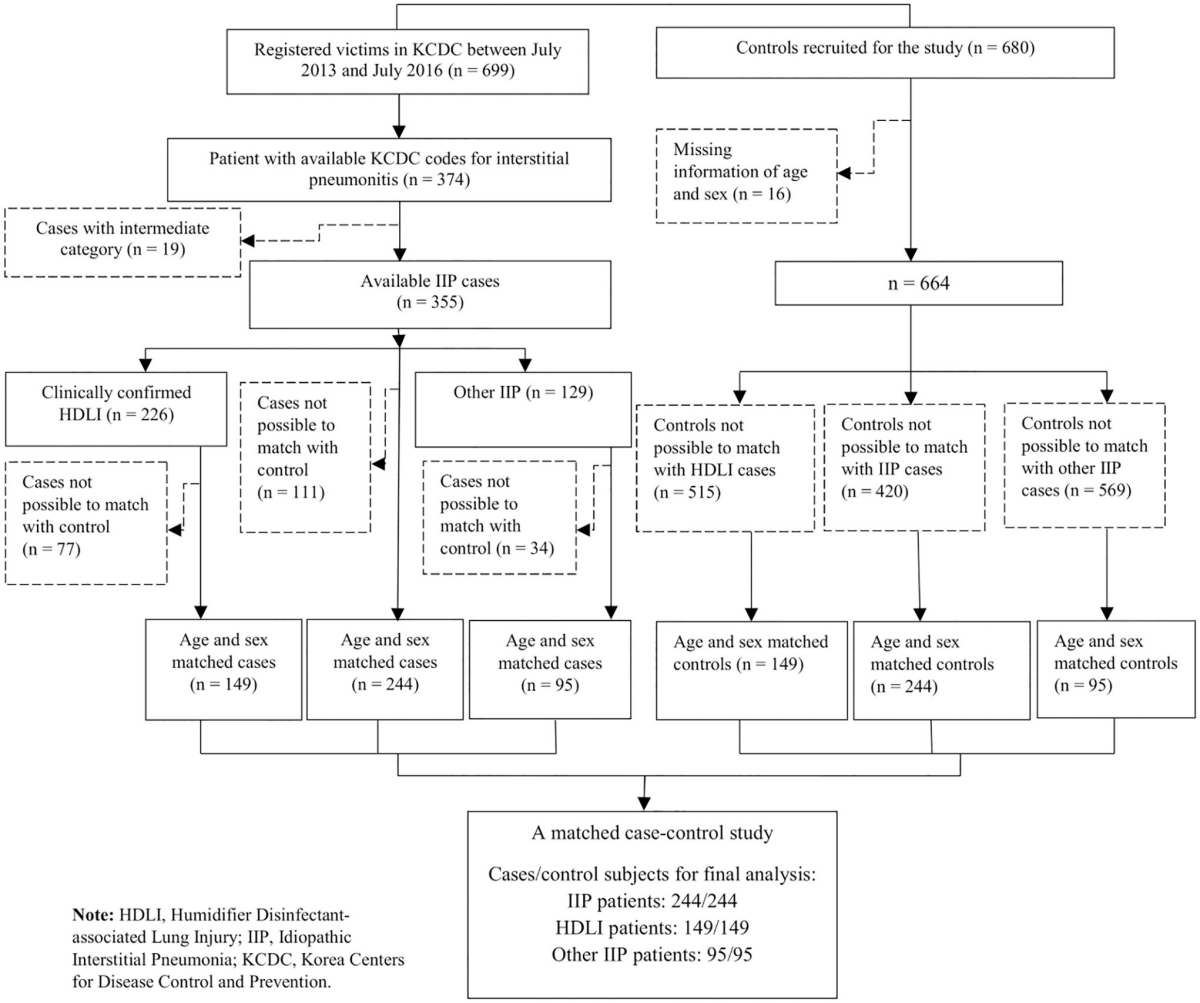
Flow chart shows selection process of case with interstitial pneumonitis and control subjects.

For the current study, we selected subjects from the first three investigations that took place from July 2013 to July 2016. Among 699 self-reported victims registered with the national program up to the third round of investigations (first round = 361; second = 169; and third = 169), 374 registered patients with lung injury case were identified using a KCD-7 code (Fig 1). We used the cases that have information about four categories of lung injury (n = 355), and the lung injury with intermediate category was excluded (n = 19). Control subjects (n = 680) were selected from non-patient family members who lived in the same residence as the registered patients and were not clinically examined and confirmed the lung injury. Recruitment of non-patient familial group as controls was expected to have less differential recall bias and provide a more appropriate comparison group than population controls and also a cost-effective alternative [18, 19].

We assigned a random number for each family number without a diagnosis of lung injury in the database by generating random numbers between 0 and 1 that were distributed uniformly. Control subjects were randomly selected and were matched to cases by 10-year age group and sex. It is unlikely that control subjects were intentionally chosen as the sample size of the control subjects was one fold as the cases with IIP. The study protocol was approved by the Institutional Review Boards of NIER (National Institute of Environmental Research) (NIER-2017-BR-008-01). Written consents were obtained from all participants after they were fully informed of the details.

### Exposure variables

Information concerning a lifetime occupational history, demographic, and tobacco use was obtained from a computer-assisted personal interview. Subjects provided information related to HD use and potential confounders such as number of household chemical products used excluding HD and presence of factories within 1 km of residence [7]. In addition, information on average hours sleeping in a room with operating humidifier treated with disinfectant, number of HD brands, type of disinfectant, and average distance (in meters) of the bed from humidifier was also obtained. The procedures of estimating retrospective exposure of HD has previously been described [7, 20]. In brief, cumulative disinfectant use hours were defined as the product of the total years, months per year, weeks per year, days per week, and hours per day of using HD. Airborne disinfectant exposure intensity was also estimated based on disinfectant volume (ml) and the frequency with which it was added to the humidifier per day, disinfectant bulk level (μg/ml), the volume of the room (m^3^), and the degree of ventilation. Furthermore, the cumulative inhalation exposure was also constructed to express the cumulative exposure level by taking into account the quantitative estimates of disinfectant exposure intensity and cumulative disinfectant use hours.

### Statistical analysis

We examined the frequency distribution of HD exposure-related characteristics and other variables according to the case and control groups. These characteristics were reported for clinically confirmed HDLI patients and patients with other IIP. Continuous variables were expressed as mean and standard deviation (SD) and categorical values as percentages. Bivariate statistical analyses were performed using the χ^2^ test for categorical variables and Wilcoxon rank-sum test for continuous variables. Measurements of HD exposure–related variables, i.e., total disinfectant use years and average hours per day, hours sleeping in a room with an operating humidifier treated with disinfectant, cumulative disinfectant use hours, and airborne disinfectant exposure intensity and airborne disinfectant exposure levels, were divided into quartiles. The average distance of the bed from humidifier (≥ 1 m, 0.5–1 m, and < 0.5 m), the number of disinfectant brands used (1, 2, and ≥ 3), and type of humidifier disinfectant (non-guanidine versus guanidine) were categorized as used in the previous study [7].

Conditional logistic regression was used to calculate odds ratios (ORs) and 95% confidence intervals (CIs) and to investigate the association between HD exposure characteristics and IIP. Each disinfectant-related exposure metric was evaluated individually in models, and multivariate logistic regression analyses were performed post hoc to identify independent risk factors. The following potential confounders were included in adjusted OR (aOR): number of household products containing chemicals, smoking, and presence of a factory within 1 km of residence. All calculated *P* values were two sided and *P* <0.05 was considered to be statistically significant. The analyses were performed by using STATA ver. 13 (STATA Corp, College Station, Texas, USA).

## Results

Demographic and residence environment data are summarized in Table 1. A total of 488 individuals were included in the study. Of these, 244 patients were diagnosed as IIP, and 244 were taken as matched controls. The IIP mostly includes interstitial pulmonary disease with fibrosis (n = 131/244; 54%) and interstitial pulmonary disease, unspecified (n = 107/244, 44%) (data not shown). Age and sex distributions of the cases and controls were similar because of 1:1 matching. Among the cases of IIP, 149 (61.1%) were clinically confirmed as HDLI and 95 (38.9%) were diagnosed as other IIP. The average age was 33.4 years, with the mean age of 19.5 years and 54.0 years among HDLI patients and other IIP patients, respectively. The percentages of men (49.2%) and women (50.8%) in the IIP patients were approximately same; the patients were more likely to be women (53.0%) than men (47.0%) in the HDLI group and more likely to be men (54.7%) than women (45.3%) in other IIP group. No significant difference between the cases and controls with regard to the number of household chemicals and the presence of a factory within 1 km of residence was observed. However, there was notable difference between cases and controls with respect to smoking. Among all patients, more patients in the control group were current smoker compared with patients in the case group (4.1% and 0.41%, respectively).

**Table 1:** Demographic and household characteristics in the case and control.

Characteristics	All IIP			HDLI			Other IIP		
	Case	Control	*P*-value	Case	Control	*P*-value	Case	Control	*P*-value
	(n = 244)	(n = 244)		(n = 149)	(n = 149)		(n = 95)	(n = 95)	
Age									
Mean (SD)	33.37 (26.28)	33.65 (25.94)	0.905	19.50 (19.40)	19.92 (19.02)	0.850	53.96 (22.10)	53.37 (22.20)	0.855
Sex, n (%)									
Male	120 (49.18)	120 (49.18)	1.000	70 (46.98)	70 (46.98)	1.000	52 (54.74)	52 (54.74)	1.000
Female	124 (50.82)	124 (50.82)		79 (53.02)	79 (53.02)		43 (45.26)	43 (45.26)	
Smoking status, n (%)									
Never	202 (82.79)	203 (83.20)		141 (94.63)	140 (93.96)		61 (64.21)	73 (76.84)	
Former	31 (12.70)	29 (11.89)	0.026	5 (3.36)	6 (4.03)	0.809	26 (27.37)	12 (12.63)	0.001
Current	1 (0.41)	10 (4.10)		1 (0.67)	2 (1.34)		0	7 (7.37)	
Missing	10 (4.10)	2 (0.82)		2 (1.34)	1 (0.67)		8 (8.42)	3 (3.16)	
No. of household products containing chemicals, n (%)									
Q1 (<2)	54 (22.13)	72 (29.51)		50 (33.56)	62 (41.61)		24 (25.26)	29 (30.53)	
Q2 (3–4)	75 (30.74)	67 (27.46)	0.319	26 (17.45)	20 (13.42)	0.449	29 (30.53)	28 (29.47)	0.591
Q3 (5–6)	68 (27.87)	60 (24.59)		47 (31.34)	40 (26.85)		12 (12.63)	15 (15.79)	
Q4 (7–11)	42 (17.21)	42 (17.21)		26 (17.45)	27 (18.12)		26 (27.37)	19 (20.00)	
Missing, n (%)	5 (2.05)	3 (1.23)					4 (4.21)	4 (4.21)	
Presence of factory within 1 km of residence, n (%)									
No	232 (95.08)	238 (97.54)	0.150	142 (95.30)	137 (91.95)	0.236	90 (94.74)	91 (95.79)	0.733
Yes	12 (4.92)	6 (2.46)		7 (4.70)	12 (8.05)		5 (5.26)	4 (4.21)	

Among all IIP patients, the average levels of HD-related characteristics, including average total use hours per day, cumulative use hours, cumulative sleep hours, average sleeping hours in a room with the use of humidifier, and cumulative exposure levels were significantly higher than those of controls (Table 2). However, the average levels of total years of HD use and airborne disinfectant intensity, and the number of disinfectant products used were not significantly different between cases and controls. In addition, compared with cases (46.3%), higher proportion of subjects in controls (69.3%) had maintained the average distance of the bed from humidifier farther than 1 m.

**Table 2:** Humidifier disinfectant use characteristics in the case and control.

Characteristics	All IIP			HDLI			Other IIP		
	Case	Control	*P*-value	Case	Control	*P*-value	Case	Control	*P*-value
	(n = 244)	(n = 244)		(n = 149)	(n = 149)		(n = 95)	(n = 95)	
Total years of use									
Mean (SD)	1.47 (1.48)	1.42 (1.43)	0.863	1.12 (1.17)	1.24 (1.23)	0.146	1.95 (1.65)	1.69 (1.82)	0.096
Missing, n (%)	4 (1.64)			1 (0.67)			3 (3.16)		
Average hours used per day									
Mean (SD)	13.41 (5.70)	10.92 (5.72)	< 0.001	13.68 (5.36)	11.63 (5.12)	< 0.001	13.02 (6.23)	9.16 (4.73)	< 0.001
Missing, n (%)	4 (1.64)	1 (0.41)		1 (0.67)	1 (0.67)		3 (3.16)	4 (4.22)	
Cumulative used hours									
Mean (SD)	6274.65 (7939.15)	4469.74 (5667.62)	< 0.001	5078.53 (6040.15)	4614.67 (6407.84)	0.117	7946.41 (10009.64)	3945.86 (5308.73)	< 0.001
Missing, n (%)	4 (1.64)	4 (1.64)		1 (0.67)	1 (0.67)		3 (3.16)	4 (4.22)	
Cumulative sleep hours									
Mean (SD)	4571.52 (4886.84)	3509.18 (4222.47)	0.001	3739.71 (4045.20)	3146.32 (3321.07)	0.110	5680.30 (5684.52)	3419.46 (4934.26)	< 0.001
Missing, n (%)	4 (1.64)			1 (0.67)			3 (3.16)	2 (2.11)	
Hours used during sleep									
Mean (SD)	9.88 (1.81)	8.79 (2.83)	< 0.001	10.11 (1.49)	9.14 (2.55)	< 0.001	9.49 (2.21)	7.68 (3.62)	< 0.001
Missing, n (%)	7 (2.87)	2 (0.82)		1 (0.67)	2 (1.34)		6 (4.32)	3 (3.16)	
Type of disinfectant, n (%)									
Non-guanidine	19 (7.79)	44 (18.03)	0.001	4 (2.68)	20 (13.42)	0.001	15 (15.79)	17 (17.89)	0.857
Guanidine	214 (87.70)	192 (78.69)		144 (96.64)	125 (83.89)		70 (73.68)	74 (77.89)	
Missing, n (%)	11 (4.51)	8 (3.28)		1 (0.67)	4 (2.68)		10 (10.52)	4 (4.21)	
No. of disinfectant products used, n (%)									
1	124 (50.82)	142 (58.20)		76 (51.01)	85 (57.05)		49 (51.58)	61 (64.21)	
2	71 (29.10)	62 (25.41)	0.411	47 (31.54)	47 (31.54)	0.431	23 (24.21)	21 (22.11)	0.209
≥ 3	39 (15.98)	37 (15.16)		24 (16.11)	17 (11.41)		15 (15.79)	9 (9.47)	
Missing, n (%)	10 (4.10)	3 (1.23)		2 (1.34)			8 (8.42)	4 (4.21)	
Average distance of the bed from humidifier, meter, n (%)									
>1 m	113 (46.31)	169 (69.26)		78 (52.35)	106 (71.14)		36 (37.89)	71 (74.74)	
0.5–1 m	89 (36.48)	61 (25.0)	< 0.001	53 (17.27)	31 (20.81)	0.004	35 (36.84)	17 (17.89)	< 0.001
< 0.5 m	36 (14.75)	11 (4.51)		17 (11.41)	12 (8.05)		19 (20.0)	4 (4.21)	
Missing, n (%)	6 (2.46)	3 (1.23)		1 (0.67)			5 (5.26)	3 (3.16)	
Air concentration, (μg/m^3^)									
Mean (SD)	128.89 (264.85)	159.29 (364.82)	0.398	151.64 (325.15)	120.19 (260.05)	0.025	88.78 (81.59)	184.52 (455.11)	0.298
Missing, n (%)	12 (4.92)	11 (4.51)		2 (1.34)	5 (3.36)		10 (10.53)	8 (8.42)	
Cumulative exposure level, unit-less									
Mean (SD)	655172.0 (1371207.0)	467251.0 (802596.3)	< 0.001	650730.40 (1480952.0)	395131.50 (594412.50)	0.003	648765.70 (1170100.00)	394175.40 (703869.8.80)	0.017
Missing, n (%)	12 (4.92)	14 (5.74)		2 (1.34)	5 (3.36)		10 (10.53)	8 (8.42)	

The association between risk of IIP and various exposure metrics describing HD use are described in Table 3. Among all patients, the risk of IIP increased approximately two-fold or more in the highest compared with the lowest quartile of average total use hours per day (aOR = 4.86, 95% CI = 2.48–9.54), cumulative sleep hours (aOR = 1.78, 95% CI = 1.02–3.10), hours sleeping in a room with an operating humidifier treated with disinfectant (aOR = 2.49, 95% CI = 1.54–4.02), and cumulative exposure level (aOR = 2.12, 95% CI = 1.14–3.94), with evidence of positive dose response and significant *P* trend for average total use hours (*P* < 0.001) and cumulative exposure level (*P* = 0.029). We also found that type of disinfectant (guanidine versus non-guanidine: aOR = 3.34, 95% CI = 1.53–7.28) and average distance of the bed from humidifier (< 5 m versus > 1 m: aOR = 5.98, 95% CI = 2.52–14.21; *P* trend < 0.001) significantly increased the risk of IIP.

**Table 3:** Conditional logistic regression for the associations of humidifier disinfectant use characteristics and idiopathic interstitial pneumonia.

Characteristics	Case	Control	Crude	Adjusted*	*P*-trend
	(n = 244)	(n = 244)	OR (95% CI)	OR (95% CI)	
Total years of use, n (%)					
Q1 (≤ 0.47)	60 (24.59)	61 (25.00)	Ref.	Ref.	
Q2 (0.48–0.94)	59 (24.18)	62 (25.41)	0.99 (0.59–1.65)	0.83 (0.48–1.43)	
Q3 (0.95–2.00)	61 (25.00)	65 (26.64)	0.99 (0.60–1.62)	0.77 (0.45–1.33)	
Q4 (2.01–8.42)	60 (24.59)	56 (22.95)	1.12 (0.66–1.92)	0.82 (0.46–1.47)	0.820
Average hours used per day, n (%)					
Q1 (≤ 8)	33 (13.52)	91 (37.30)	Ref.	Ref.	
Q2 (9–11)	83 (34.02)	73 (29.92)	3.55 (2.00–6.30)	3.26 (1.78–5.96)	
Q3 (12–13)	53 (21.72)	33 (13.52)	6.45 (3.12–13.33)	5.87 (2.74–12.57)	
Q4 (14–24)	71 (29.10)	46 (18.85)	5.20 (2.74–9.88)	4.86 (2.48–9.54)	< 0.001
Cumulative use hours, n (%)					
Q1 (≤ 1344)	51 (20.90)	71 (29.10)	Ref.	Ref.	
Q2 (1345–3024)	60 (24.59)	59 (24.18)	1.43 (0.84–2.45)	1.23 (0.70–2.16)	
Q3 (3025–7056)	58 (23.77)	62 (25.41)	1.34 (0.81–2.20)	1.06 (0.62–1.80)	
Q4 (7057–67872)	71 (29.10)	48 (19.67)	2.37 (1.35–4.15)	1.81 (0.99–3.30)^§^	0.215
Cumulative sleep hours, n (%)					
Q1 (≤ 1078)	48 (19.67)	73 (29.92)	Ref.	Ref.	
Q2 (1079–2352)	63 (25.82)	58 (23.77)	1.64 (0.96–2.80)^§^	1.45 (0.83–2.55)	
Q3 (2353–4866.4)	56 (22.95)	65 (26.64)	1.35 (0.80–2.27)	1.07 (0.61–1.86)	
Q4 (4866.5–31108)	73 (29.92)	48 (19.67)	2.35 (1.39–3.98)	1.78 (1.02–3.10)	0.117
Hours used during sleep, n (%)					
Q1 (≤ 8)	43 (17.62)	89 (36.48)	Ref.	Ref.	
Q2 (9–11)	194 (79.51)	153 (62.70)	2.69 (1.72–4.22)	2.49 (1.54–4.02)	
Type of disinfectant, n (%)					
Non-guanidine	19 (7.79)	44 (18.03)	Ref.	Ref.	
Guanidine	214 (87.70)	192 (78.69)	3.44 (1.64–7.23)	3.34 (1.53–7.28)	
No. of disinfectant products used, n (%)					
1	124 (50.82)	142 (58.20)	Ref.	Ref.	
2	71 (29.10)	62 (25.41)	1.33 (0.88–2.02)	1.35 (0.87–2.09)	
≥ 3	39 (15.98)	37 (15.16)	1.21 (0.72–2.03)	1.08 (0.53–1.85)	0.408
Average distance of the bed from humidifier, meter, n (%)					
>1 m	113 (46.31)	169 (69.26)	Ref.	Ref.	
0.5–1 m	89 (36.48)	61 (25.00)	2.24 (1.44–3.48)	2.28 (1.44–3.60)	
< 0.5 m	36 (14.75)	11 (4.51)	5.32 (2.34–11.83)	5.98 (2.52–14.21)	< 0.001
Air concentration, (μg/m^3^), n (%)					
Q1 (≤ 40.0)	54 (22.13)	64 (26.23)	Ref.	Ref.	
Q2 (40.1–77.3)	59 (24.18)	56 (22.95)	1.19 (0.70–2.02)	1.23 (0.71–2.15)	
Q3 (77.4–148.5)	60 (24.59)	56 (22.95)	1.21 (0.71–2.07)	1.10 (0.64–1.91)	
Q4 (148.6–3805.3)	59 (24.18)	57 (23.36)	1.10 (0.66–1.84)	1.11 (0.65–1.89)	0.901
Cumulative exposure level, unit-less, n (%)					
Q1 (≤ 72141.7)	41 (16.80)	75 (30.74)	Ref.	Ref.	
Q2 (72141.8–227595.1)	58 (23.77)	57 (23.36)	1.82 (1.02–3.25)	1.75 (0.96–3.19)^§^	
Q3 (227595.2–598119.4)	67 (27.46)	49 (20.08)	2.60 (1.46–4.64)	2.42 (1.33–4.39)	
Q4 (598119.5–1 x 10^7^)	66 (27.05)	49 (20.08)	2.67 (1.48–4.81)	2.12 (1.14–3.94)	0.029

Total numbers may not be equal to the total case and control numbers for some characteristics due to missing data.

*Adjusted for smoking status, number of chemicals used in house, and presence of factory around house.

^§^*P* = 0.05-< 0.10.

Cumulative use hours and inhaled HD concentration were not significantly associated with the risk of IIP (Table 3). However, the elevated level of inhaled HD concentration was associated with HDLI risk (Q_4_ versus Q_1_: aOR = 3.01, 95% CI = 1.34–6.76) (Table 4). In contrast, there were no significant associations between the high level of the HD concentration and other IIP (Table 5). In addition, cumulative exposure levels were not significantly associated with other IIP. The cumulative use hours of HD showed no significant association with HDLI risk, but was significantly associated with other IIP (Tables 4 and 5).

**Table 4:** Conditional logistic regression for the associations of humidifier disinfectant use characteristics and HDLI.

Characteristics	Case	Control	Crude	Adjusted*	*P*-trend
	(n = 149)	(n = 149)	OR (95% CI)	OR (95% CI)	
Total years of use, n (%)					
Q1 (≤ 0.47)	44 (29.53)	38 (25.50)	Ref.	Ref.	
Q2 (0.48–0.94)	43 (28.86)	43 (28.86)	0.83 (0.45–1.54)	0.89 (0.45–1.74)	
Q3 (0.95–2.00)	40 (26.85)	43 (28.86)	0.76 (0.41–1.43)	0.87 (0.45–1.69)	
Q4 (2.01–8.42)	21 (14.09)	25 (16.78)	0.67 (0.31–1.46)	0.74 (0.33–1.64)	0.352
Average hours used per day, n (%)					
Q1 (≤ 8)	15 (10.07)	40 (26.85)	Ref.	Ref.	
Q2 (9–11)	46 (30.87)	46 (30.87)	2.68 (1.27–5.66)	2.84 (1.29–6.24)	
Q3 (12–13)	44 (29.53)	31 (20.81)	3.73 (1.70–8.22)	3.53 (1.56–8.00)	
Q4 (14–24)	43 (28.86)	31 (20.81)	3.66 (1.66–8.06)	3.96 (1.71–9.16)	0.001
Cumulative use hours, n (%)					
Q1 (≤ 1344)	31 (20.81)	36 (24.16)	Ref.	Ref.	
Q2 (1345–3024)	47 (31.54)	53 (35.57)	1.05 (0.57–1.92)	1.09 (0.57–2.09)	
Q3 (3025–7056)	37 (24.83)	36 (24.16)	1.13 (0.60–2.13)	1.45 (0.74–2.84)	
Q4 (7057–67872)	33 (22.15)	23 (15.44)	1.72 (0.80–3.70)	1.89 (0.86–4.17)	0.145
Cumulative sleep hours, n (%)					
Q1 (≤ 1078)	29 (19.46)	37 (24.83)	Ref.	Ref.	
Q2 (1079–2352)	48 (32.21)	45 (30.20)	1.34 (0.73–2.48)	1.50 (0.78–2.89)	
Q3 (2353–4866.4)	37 (24.83)	39 (26.17)	1.17 (0.62–2.20)	1.30 (0.67–2.53)	
Q4 (4866.5–31108)	34 (22.82)	28 (18.79)	1.52 (0.76–3.03)	1.81 (0.87–3.73)	0.310
Hours used during sleep, n (%)					
Q1 (≤ 8)	23 (15.44)	44 (29.53)	Ref.	Ref.	
Q2 (9–11)	125 (83.89)	103 (69.13)	2.47 (1.35–4.49)	2.52 (1.36–4.68)	
Type of disinfectant, n (%)					
Non-guanidine	4 (2.68)	20 (13.42)	Ref.	Ref.	
Guanidine	144 (96.64)	125 (83.89)	6.33 (1.87–21.40)	9.77 (2.38–40.18)	
No. of disinfectant products used, n (%)					
1	76 (51.01)	85 (57.05)	Ref.	Ref.	
2	47 (31.54)	47 (31.54)	1.12 (0.67–1.87)	1.20 (0.69–2.09)	
≥ 3	24 (16.11)	17 (11.41)	1.59 (0.77–3.26)	1.85 (0.86–3.98)	0.284
Average distance of the bed from humidifier, meter, n (%)					
>1 m	78 (52.35)	106 (71.14)	Ref.	Ref.	
0.5–1 m	53 (35.57)	31 (20.81)	2.37 (1.36–4.15)	2.41 (1.35–4.30)	
< 0.5 m	17 (11.41)	12 (8.05)	1.73 (0.77–3.91)	1.70 (0.72–3.99)	0.075
Air concentration (μg/m^3^), n (%)					
Q1 (≤ 40.0)	27 (18.12)	34 (22.82)	Ref.	Ref.	
Q2 (40.1–77.3)	40 (26.85)	42 (28.19)	1.16 (0.60–2.24)	1.30 (0.65–2.62)	
Q3 (77.4–148.5)	36 (24.16)	44 (29.53)	1.01 (0.50–2.02)	1.05 (0.50–2.20)	
Q4 (148.6–3805.3)	44 (29.53)	24 (16.11)	2.26 (1.08–4.71)	3.01 (1.34–6.76)	0.105
Cumulative exposure level, unit-less, n (%)					
Q1 (≤ 72141.7)	23 (14.44)	37 (24.83)	Ref.	Ref.	
Q2 (72141.8–227595.1)	38 (25.50)	43 (28.86)	1.16 (0.56–2.44)	1.39 (0.61–3.16)	
Q3 (227595.2–598119.4)	45 (30.20)	41 (27.52)	1.50 (0.77–2.92)	1.84 (0.88–3.82)^§^	
Q4 (598119.5–1 x 10^7^)	41 (27.52)	23 (15.44)	2.82 (1.33–5.97)	3.57 (1.59–8.01)	0.003

Total numbers may not be equal to the total case and control numbers for some characteristics due to missing data.

*Adjusted for smoking status, number of chemicals used in house, and presence of factory around house.

^§^*P* = 0.05 − < 0.10.

**Table 5:** Conditional logistic regression for the associations of humidifier disinfectant use characteristics and other idiopathic interstitial pneumonia.

Characteristics	Case	Control	Crude	Adjusted*	*P*-trend
	(n = 95)	(n = 95)	OR (95% CI)	OR (95% CI)	
Total years of use, n (%)					
Q1 (≤ 0.47)	17 (17.89)	27 (28.42)	Ref.	Ref.	
Q2 (0.48–0.94)	15 (15.79)	24 (25.26)	0.83 (0.35–1.99)	0.61 (0.21–1.80)	
Q3 (0.95–2.00)	22 (23.16)	16 (16.84)	1.82 (0.80–4.16)	1.72 (0.68–4.34)	
Q4 (2.01–8.42)	38 (40.00)	28 (29.47)	2.07 (0.94–4.56)^§^	1.87 (0.72–4.87)	0.287
Average hours used per day, n (%)					
Q1 (≤ 8)	18 (18.95)	41 (43.16)	Ref.	Ref.	
Q2 (9–11)	37 (38.95)	20 (21.05)	4.09 (1.60–10.46)	4.54 (1.63–12.65)	
Q3 (12–13)	9 (9.47)	24 (25.26)	0.58 (0.14–2.33)	0.52 (0.09–3.17)	
Q4 (14–24)	28 (29.47)	6 (6.32)	10.95 (2.93–40.94)	9.07 (2.14–38.44)	<0.001
Cumulative use hours, n (%)					
Q1 (≤ 1344)	22 (23.16)	39 (41.05)	Ref.	Ref.	
Q2 (1345–3024)	13 (13.68)	15 (15.79)	1.31 (0.53–3.28)	1.25 (0.44–3.51)	
Q3 (3025–7056)	20 (21.05)	18 (18.95)	1.53 (0.70–3.34)	1.61 (0.67–3.87)	
Q4 (7057–67872)	37 (38.95)	19 (20.00)	3.51 (1.47–8.39)	3.77 (1.35–10.54)	0.001
Cumulative sleep hours, n (%)					
Q1 (≤ 1078)	19 (20.00)	39 (41.05)	Ref.	Ref.	
Q2 (1079–2352)	17 (17.89)	16 (16.84)	2.07 (0.83–5.17)	2.15 (0.74–6.19)	
Q3 (2353–4866.4)	18 (18.95)	20 (21.05)	1.45 (0.63–3.36)	1.14 (0.42–3.06)	
Q4 (4866.5–31108)	38 (40.00)	18 (18.95)	4.75 (1.89–11.97)	5.39 (1.81–16.03)	0.090
Hours used during sleep, n (%)					
Q1 (≤ 8)	20 (21.05)	43 (45.26)	Ref	Ref.	
Q2 (9–11)	69 (72.63)	49 (51.58)	3.09 (1.57–6.10)	3.23 (1.47–7.10)	
Type of disinfectant					
Non-guanidine	15 (15.79)	17 (17.89)	Ref.	Ref.	
Guanidine	70 (73.68)	74 (77.89)	1.17 (0.54–2.52)	1.10 (0.47–2.57)	
No. of disinfectant products used, n (%)					
1	49 (51.58)	61 (64.21)	Ref.	Ref.	
2	23 (24.21)	21 (22.11)	1.24 (0.64–2.39)	1.30 (0.61–2.76)	
≥ 3	15 (15.79)	9 (9.47)	1.98 (0.77–5.09)	1.73 (0.63–4.76)	0.518
Average distance of the bed from humidifier, meter, n (%)					
>1 m	36 (37.89)	71 (74.74)	Ref.	Ref.	
0.5–1 m	35 (36.84)	17 (17.89)	4.43 (1.91–10.25)	4.04 (1.62–10.09)	
< 0.5 m	19 (20.00)	4 (4.21)	7.86 (2.16–28.34)	11.38 (2.33–55.66)	< 0.001
Air concentration (μg/m^3^), n (%)					
Q1 (≤ 40.0)	28 (29.47)	22 (23.16)	Ref.	Ref.	
Q2 (40.1–77.3)	19 (20.00)	24 (25.26)	0.69 (0.31–1.51)	0.55 (0.22–1.36)	
Q3 (77.4–148.5)	23 (24.21)	19 (20.00)	0.94 (0.35–2.57)	0.82 (0.27–2.50)	
Q4 (148.6–3805.3)	15 (15.79)	22 (23.16)	0.57 (0.23–1.40)	0.42 (0.15–1.17)	0.300
Cumulative exposure level, unit-less, n (%)					
Q1 (≤ 72141.7)	20 (21.05)	29 (30.53)	Ref.	Ref.	
Q2 (72141.8–227595.1)	21 (22.11)	23 (24.21)	1.14 (0.50–2.64)	1.06 (0.43–2.63)	
Q3 (227595.2–598119.4)	19 (20.00)	19 (20.00)	1.31 (0.54–3.16)	1.31 (0.51–3.37)	
Q4 (598119.5–1 x 10^7^)	25 (26.32)	16 (16.84)	1.60 (0.68–3.77)	1.63 (0.62–4.30)	0.062

Total numbers may not be equal to the total case and control numbers for some characteristics due to missing data.

*Adjusted for smoking status, number of chemicals used in house, and presence of factory around house.

^§^*P* = 0.05 − < 0.10.

Odds ratios for average total use hours per day, cumulative sleep hours, and average distance of the bed apart from the humidifier were higher among patients with other IIP. For example, the odds ratio for the average distance of the bed apart from humidifier was 11.38 (95% CI = 2.33–55.66; < 0.5 m versus > 1 m) in the patients with other IIP group compared with 1.70 (95% CI = 0.72–3.99; <0.5 m versus > 1 m) among patients with HDLI group (Tables 4 and 5).

## Discussion

Our findings support and expand those of previous case-control studies [7, 9, 15] reporting increased risk for HDLI associated with several HD use characteristics; other IIP were also found to be significantly associated with the HD use characteristics. This is the first case-control study to provide evidence of relationship between HD exposure metrics and IIP other than HDLI in this population.

We found that inhaled HD concentration (μg/m^3^), which was estimated by average HD amount and hours of HD use/day (mL), room size (m^3^), concentration of chemicals, and ventilation rate, was significantly associated with HDLI risk, but not for the other IIP. This discrepancy is most likely caused by the differences in associations between the HD concentration, duration of HD use, and the age of the study subjects. To test the possibility of whether age and duration of HD use were associated with IIP risk, we constructed a new model for the duration of HD (dichotomized by the median distribution) with interaction with age (dichotomized by the median distribution), using all patients (n = 488). This model, after adjustment for sex, presence of factory, and smoking status, showed a significant interaction between the duration of HD use and age (*P* = 0.021) (data not shown). This indicates that HDLI patients with different duration of HD exposure and age differ in the rate of the pneumonitis risk. The average age of HDLI patients (19.5 years) was lower than that of other IIP patients (54.0 years). In our study population, HDLI victims were largely distributed to younger age group. This finding further adds to the evidence that younger people was more vulnerable to lung damage when exposed to HDs [9]. Likewise, the cumulative HD inhalation exposure showed significant association with HDLI risk, but not associated with other IIP.

Cumulative HD use hours that provide a measure of chronic exposure were not related to the risk of HDLI, but was related to other IIP. The possible reasons for this relationship may be due to the different patterns of HD use or may be related to the age of study subjects. Park et al. (2015) indicated that insignificant findings of cumulative HD use hours may be related to the pattern of HDs use, such as some people used HDs intermittently during specific events or reasons, while other used HD continuously [7]. Further, in our study, the average level of cumulative HD use in HDLI patients was lower (mean = 5078.53) compared to other IIP patients (mean = 8797.10) (Table 2). When cumulative HD use hours were utilized to compare group differences in exposure, which revealed significant difference between age groups (19.5 years versus 54.0 years; Wilcoxon rank-sum test, *P* = 0.029).

Type of disinfectant was found to be strongly associated with HDLI risk but not for the risk of other IIP. Those using humidifier brands containing the guanidine disinfectant chemical group (PGH or PHMG) showed a higher risk of HDLI compared to those using brands containing non-guanidine (a mixture of CMIT/MIT). In our study, the number of HDLI patients who used HD brands containing PGH and PHMG (87.7%) was far higher than those used non-guanidine chemical group (7.8%) (Table 2). Previous study reported that the use of guanidine disinfectant chemical was associated with the risk of HDLI in South Korea [21]. However, this study was done only on HDLI patients. Difference in strength of associations between the HDLI and other pneumonitis could be explained by the age and length of HD use. Park et al. reported that the type of HD exhibited different levels of risk of development of HDLI according to age, pregnancy, and length of HD use [8]. They reported that, among PHMG user group in a home, patients aged ≤ 6 years had the highest number of deaths among HDLI group, but in the other IIP group, the highest number of death was recorded for the patients aged ≥19 years. The active ingredient of guanidine group includes PHMG and PGH and the ingredient for non-guanidine group includes the mixture of CMIT/MIT. It is possible that these ingredients might produce a mixed effect, such as synergistic or additive effects, among HDLI patients who used multiple HD products. Further studies are needed to investigate the possibility of mixed effects between guanidine and non-guanidine chemicals.

Biological plausibility of our results based on the ingredients of HD products containing guanidine and non-guanidine chemical groups confirmed by experimental toxicological studies on animals [5, 22], in addition to known clinical effects of HD exposure [6]. The ultrasonic humidifier usually operated with tap water and may release dissolved particles with nanometers in size [23]. The smallest size distribution of submicron particles (< 1 μ m) and nanoparticles (< 100 nm) can penetrate deep into the alveolar region where removal mechanism may be insufficient [24, 25]. One possible mechanism is that dispersed fine HD particles can be used as delivery carriers to deliver the chemicals deeper into the lung, and it may cause potential damage to the cells. These particles can physically interact with tissue or cell, which may lead to physical damage to the cell or genetic material. In addition, generation of reactive oxygen species induced by PHMG phosphate has been identified as releasing fibrotic inflammatory cytokines, and trigger a wound-healing response, leading to pulmonary fibrosis [22]. In our study, PHMG was also found to be associated with IIP that also includes other interstitial pulmonary disease with fibrosis (K-J84.18). The disease code of K-J84.18 was defined as idiopathic pulmonary fibrosis (IPF) [26]. Previous study reported that chronic lung injury is related to diffuse pulmonary inflammation, which may promote interstitial pulmonary fibrotic disease such as IPF [27]. The pulmonary fibrosis has been shown to be associated with exposure to several chemical [28, 29].

The findings of our study can provide useful information regarding HD exposure and HDLI risk according to the clinical examination category. The identification of HDLI cases was done by a team of experts using standardized guidelines recommend for investigation of HDLI. Further, the sample selection of cases and controls was matched according to age and gender, and the control group was selected from non-patient family members who lived in the same residence as HDLI patients, also adding to the strengths of the study findings. Nevertheless, there are several limitations. Although we used systematic approach to collect exposure data [20], the possibility of recall and subjectivity bias may remain because HDLI cases and family controls may have differential recall and awareness of the study objectives. When the cases are aware of the study objectives, it is likely to result in higher exposure reporting [30]. However, it is most probably a non-differential one between the two groups of comparison, as the study subjects were not aware of the study objectives. In addition, there is a possibility of selection bias as our study subjects are based on people who self-reported to KCDC. Many of subjects are also included at the request of the family of victims, presumably because they had noticed symptoms of lung injury. Moreover, we did not have adequate numbers to separately evaluate effects in different age groups of victims since there is limited data regarding the potential exposure levels experienced by different age groups.

In conclusion, the use of household HDs contribute are potential risk factors for IIP not only to the patients with HDLI but also to the patients with other pneumonitis. Therefore, continuous monitoring and reconsideration of clinical categorization of HDLI could be needed to include wider range of susceptible individuals.
